# Supplementary material for: Gut Microbiota Changes during Dimethyl Fumarate Treatment in Patients with Multiple Sclerosis
Source: Int J Mol Sci. 2023 Feb 1;24(3):2720. doi: 10.3390/ijms24032720 (PMC9917003; doi:10.3390/ijms24032720)
Supplement: Supplementary file 1 [file ijms-24-02720-s001.zip › ijms-2165457-supplementary.pdf]

International Journal of *Molecular Sciences*

Supplementary material

Table S1. Diet characteristics of the study population.

	**N=19**
**Diet**	
Stimated daily macronutrients assumption, g (SD)	
Proteins	66 (21.4)
Carbohydrates	230.1 (64.4)
Sugar	97.1 (30.4)
Lipids	61.7 (24.8)
SFA	21.4 (8.3)
PUFA	9.1 (3.8)
Fibers	22.8 (8.5)
Alcohol	5.7 (8.6)
Stimated daily oligoelements assumption, (SD)	
Calcium, mg	863.4 (289.8)
Iron, mg	10.6 (3.3)
Zinc, mg	9.4 (2.9)
Vitamin A, mcg	1131.3 (577.7)
Vitamin D, mcg	1.7 (1)
Vitamin E, mg	10.5 (4.8)
Folic acid, mcg	338.4 (125.6)
Vitamin B12, mcg	3.5 (1.6)
Vitamin C, mg	174.8 (83.9)
Percentage of daily Kcal, (SD)	
Carbohydrates	50.2 (5.2)
Sugar	21.5 (5.9)
Lipid	32.1 (5.4)
SFA	10.9 (2.3)
PUFA	4.8 (1.5)

SD, standard deviation; SFA, short chain fatty acid; PUFA, polyunsaturated fatty acid.

Table S2. Relative abundance of the gut microbiota *Bifidobacterium* species and subspecies during the treatment with DMF.

Bifidobacteria							
B adolescentis	0.47678196 (0.306721145)	0.42554148 (0.240353978)	*0.157*	0.42750554 (0.255919597)	*0.629*	0.40737818 (0.317212411)	*0.868*
B animalis	0.02099287 (0.042715600)	0.03651788 (0.090415646)	*0.272*	0.05105949 (0.145787709)	*0.125*	0.04424180 (0.126915525)	*0.552*
B asteroides	0.01291550 (0.020704177)	0.01075352 (0.014353747)	*0.929*	0.01563825 (0.020413285)	*0.445*	0.00884782 (0.014292040)	*0.508*
B bifidum	0.02174961 (0.025978833)	0.04263239 (0.070240133)	*0.408*	0.02020020 (0.028942614)	*0.352*	0.03155854 (0.045401002)	*0.198*
B breve	0.03711894 (0.055211414)	0.04029022 (0.041673122)	*0.875*	0.05561245 (0.076665033)	*0.125*	0.03617707 (0.051669218)	*0.382*
B dentium	0.04787519 (0.073482547)	0.05965765 (0.102063457)	*0.826*	0.04595015 (0.062768742)	*0.717*	0.08774106 (0.185822868)	*0.925*
B longum subsp. longum	0.23073736 (0.185914226)	0.28920111 (0.217876067)	*0.133*	0.25308719 (0.157490527)	*0.520*	0.24725105 (0.158400990)	*0.463*
B pseudocatenulatum	0.03858645 (0.095749212)	0.02390096 (0.040115942)	*0.753*	0.01938056 (0.043061207)	*0.554*	0.03859296 (0.070546908)	*0.753*
B pseudolongum	0.02520292 (0.035811507)	0.03101052 (0.038189016)	*0.534*	0.04153004 (0.057684266)	*0.074*	0.032570418623189 (0.047058737496740)	*0.799*

SD, standard deviation; *B*., *Bifidobacterium*.
[a] Wilcoxon test.

Table S3. List of analyzed taxa with abundance over 1%.

**Phylum**	**Class**	**Order**	**Family**	**Genus**
Actinobacteria*	Actinobacteria	Bifidobacteriales	Bifidobacteriaceae*	*Bifidobacterium**
Bacteroidetes*	Bacteroidia	Bacteroidales	Bacteroidaceae*	*Bacteroides**
	Bacteroidia	Bacteroidales	Barnesiellaceae*	*Barnesiella**
	Bacteroidia	Bacteroidales	Barnesiellaceae	*Coprobacter*
	Bacteroidia	Bacteroidales	Barnesiellaceae	uncultured
	Bacteroidia	Bacteroidales	Marinifilaceae*	*Butyricimonas**
	Bacteroidia	Bacteroidales	Marinifilaceae	*Odoribacter**
	Bacteroidia	Bacteroidales	Muribaculaceae	uncultured
	Bacteroidia	Bacteroidales	Prevotellaceae*	*Alloprevotella*
	Bacteroidia	Bacteroidales	Prevotellaceae	*Paraprevotella**
	Bacteroidia	Bacteroidales	Prevotellaceae	*Prevotella* 2
	Bacteroidia	Bacteroidales	Prevotellaceae	*Prevotella* 7
	Bacteroidia	Bacteroidales	Prevotellaceae	*Prevotella* 9*
	Bacteroidia	Bacteroidales	Prevotellaceae	*Prevotella* sp. AN 5135
	Bacteroidia	Bacteroidales	Prevotellaceae	Prevotellaceae NK3B31 group
	Bacteroidia	Bacteroidales	Prevotellaceae	Prevotellaceae UCG-001

	Bacteroidia	Bacteroidales	Prevotellaceae	Prevotellaceae UCG-004
	Bacteroidia	Bacteroidales	Prevotellaceae	uncultered
	Bacteroidia	Bacteroidales	Rikenellaceae*	*Alistipes**
	Bacteroidia	Bacteroidales	Tannerellaceae*	*Parabacteroides**
Cyanobacteria*	Melainabacteria	Gastranaerophilales	Acinetobacter sp. CAG:196	Acinetobacter sp. CAG:196
	Melainabacteria	Gastranaerophilales	uncultured	uncultured
Firmicutes*	Bacilli	Lactobacillales	Streptococcaceae*	*Streptococcus**
	Clostridia	Clostridiales	Christensenellaceae*	Christensenellaceae R-7 group*
	Clostridia	Clostridiales	Clostridiaceae 1*	*Clostridium* sensu stricto 1*
	Clostridia	Clostridiales	Clostridiales vadinBB60 group*	Clostridiales bacterium feline oral taxon 148
	Clostridia	Clostridiales	Lachnospiraceae*	*Eubacterium eligens* group*
	Clostridia	Clostridiales	Lachnospiraceae	*Eubacterium ruminantium* group
	Clostridia	Clostridiales	Lachnospiraceae	*Eubacterium ventriosum* group
	Clostridia	Clostridiales	Lachnospiraceae	*Eubacterium xylanophilum* group
	Clostridia	Clostridiales	Lachnospiraceae	*Ruminococcus gnavus* group
	Clostridia	Clostridiales	Lachnospiraceae	*Agathobacter**
	Clostridia	Clostridiales	Lachnospiraceae	*Anaerostipes**
	Clostridia	Clostridiales	Lachnospiraceae	*Blautia**
	Clostridia	Clostridiales	Lachnospiraceae	*Cellulosilyticum*
	Clostridia	Clostridiales	Lachnospiraceae	*Coprococcus* 1*
	Clostridia	Clostridiales	Lachnospiraceae	*Coprococcus* 2
	Clostridia	Clostridiales	Lachnospiraceae	*Coprococcus* 3
	Clostridia	Clostridiales	Lachnospiraceae	*Dorea**
	Clostridia	Clostridiales	Lachnospiraceae	*Eisenbergiella*
	Clostridia	Clostridiales	Lachnospiraceae	*Fusicatenibacter*
	Clostridia	Clostridiales	Lachnospiraceae	*Lachnoclostridium**
	Clostridia	Clostridiales	Lachnospiraceae	*Lachnospira**
	Clostridia	Clostridiales	Lachnospiraceae	Lachnospiraceae ND3007 group
	Clostridia	Clostridiales	Lachnospiraceae	Lachnospiraceae NK4A136 group*
	Clostridia	Clostridiales	Lachnospiraceae	Lachnospiraceae UCG-004
	Clostridia	Clostridiales	Lachnospiraceae	Lachnospiraceae UCG-007
	Clostridia	Clostridiales	Lachnospiraceae	Lachnospiraceae UCG-010
	Clostridia	Clostridiales	Lachnospiraceae	*Marvinbryantia*
	Clostridia	Clostridiales	Lachnospiraceae	*Pseudobutyrivibrio*
	Clostridia	Clostridiales	Lachnospiraceae	*Roseburia**
	Clostridia	Clostridiales	Lachnospiraceae	*Shuttleworthia*

	Clostridia	Clostridiales	Lachnospiraceae	*Tyzzerella* 4
	Clostridia	Clostridiales	Peptostreptococcaceae	*Romboutsia*
	Clostridia	Clostridiales	Ruminococcaceae*	*Eubacterium coprostanoligenes* group*
	Clostridia	Clostridiales	Ruminococcaceae	*Butyricicoccus*
	Clostridia	Clostridiales	Ruminococcaceae	CAG-352
	Clostridia	Clostridiales	Ruminococcaceae	*Faecalibacterium**
	Clostridia	Clostridiales	Ruminococcaceae	*Flavonifractor*
	Clostridia	Clostridiales	Ruminococcaceae	*Negativibacillus*
	Clostridia	Clostridiales	Ruminococcaceae	*Oscillibacter*
	Clostridia	Clostridiales	Ruminococcaceae	*Oscillospira*
	Clostridia	Clostridiales	Ruminococcaceae	*Ruminiclostridium* 5
	Clostridia	Clostridiales	Ruminococcaceae	*Ruminiclostridium* 6
	Clostridia	Clostridiales	Ruminococcaceae	*Ruminiclostridium* 9
	Clostridia	Clostridiales	Ruminococcaceae	Ruminococcaceae NK4A214 group*
	Clostridia	Clostridiales	Ruminococcaceae	Ruminococcaceae UCG-002*
	Clostridia	Clostridiales	Ruminococcaceae	Ruminococcaceae UCG-003
	Clostridia	Clostridiales	Ruminococcaceae	Ruminococcaceae UCG-005
	Clostridia	Clostridiales	Ruminococcaceae	Ruminococcaceae UCG-010
	Clostridia	Clostridiales	Ruminococcaceae	Ruminococcaceae UCG-014*
	Clostridia	Clostridiales	Ruminococcaceae	*Ruminococcus* 1*
	Clostridia	Clostridiales	Ruminococcaceae	*Ruminococcus* 2*
	Clostridia	Clostridiales	Ruminococcaceae	*Subdoligranulum**
	Clostridia	Clostridiales	Ruminococcaceae	uncultured
	Erysipelotrichia	Erysipelotrichales	Erysipelotrichaceae	*Asteroleplasma*
	Erysipelotrichia	Erysipelotrichales	Erysipelotrichaceae	*Catenibacterium*
	Erysipelotrichia	Erysipelotrichales	Erysipelotrichaceae	*Coprobacillus*
	Erysipelotrichia	Erysipelotrichales	Erysipelotrichaceae	*Holdemanella*
	Negativicutes	Selenomonadales	Acidaminococcaceae*	*Acidaminococcus*
	Negativicutes	Selenomonadales	Acidaminococcaceae	*Phascolarctobacterium*
	Negativicutes	Selenomonadales	Acidaminococcaceae	*Succiniclasticum*
	Negativicutes	Selenomonadales	Veillonellaceae*	*Dialister*
	Negativicutes	Selenomonadales	Veillonellaceae	*Megasphaera*
	Negativicutes	Selenomonadales	Veillonellaceae	*Negativicoccus*
	Negativicutes	Selenomonadales	Veillonellaceae	*Veillonella*
Proteobacteria*	Alphaproteobacteria	Rhodospirillales	uncultured	*Azospirillum* sp. 47_25
	Alphaproteobacteria	Rhodospirillales	uncultured	uncultured
	Deltaproteobacteria	Desulfovibrionales	Desulfovibrionaceae*	
	Gammaproteobacteria	Aeromonadales	Succinivibrionaceae*	*Succinivibrio*
	Gammaproteobacteria	Betaproteobacteriales	Burkholderiaceae*	*Parasutterella*
	Gammaproteobacteria	Betaproteobacteriales	Burkholderiaceae	*Sutterella**

	Gammaproteobacteria	Enterobacteriales	Enterobacteriaceae*	*Escherichia-Shigella*
	Gammaproteobacteria	Enterobacteriales	Enterobacteriaceae	*Klebsiella*
	Gammaproteobacteria	Enterobacteriales	Enterobacteriaceae	*Pseudocitrobacter*
	Gammaproteobacteria	Pasteurellales	Pasteurellaceae*	*Haemophilus**
Tenericutes*	Mollicutes	Izimaplasmatales	uncultured	uncultured
	Mollicutes	Mollicutes RF39	Firmicutes bacterium CAG:822	uncultured
Verrucomicrobia*	Verrucomicrobiae	Verrucomicrobiales	Akkermansiaceae*	*Akkermansia**

*taxa of particular interest which were included in the statistical analysis (most abundant or most studied or suggested to be influenced by DMTs).
